# Experimental infection of high health pigs with porcine circovirus type 2 (PCV2) and *Lawsonia intracellularis*

**DOI:** 10.3389/fvets.2022.994147

**Published:** 2022-10-06

**Authors:** Mette S. Hansen, Tim K. Jensen, Charlotte K. Hjulsager, Øystein Angen, Ulla Riber, Jens Nielsen, Peter M. H. Heegaard, Lars E. Larsen

**Affiliations:** ^1^Center for Diagnostic, Technical University of Denmark (DTU), Kgs. Lyngby, Denmark; ^2^The National Veterinary Institute, DTU, Kalvehave, Denmark; ^3^Department of Veterinary and Animal Sciences, Faculty of Health and Medical Sciences, University of Copenhagen, Frederiksberg, Denmark; ^4^Statens Serum Institut, Copenhagen, Denmark; ^5^The National Veterinary Institute, DTU, Frederiksberg, Denmark; ^6^National Institute of Aquatic Resources, DTU, Kgs. Lyngby, Denmark; ^7^Experimental and Translational Immunology, Department of Health Technology, DTU, Kgs. Lyngby, Denmark

**Keywords:** porcine circovirus type 2(PCV2), experimental infection, delayed co-infection model, porcine enteritis, Lawsonia intracellularis

## Abstract

**Background:**

Porcine circovirus type 2 (PCV2) and *Lawsonia intracellularis* infections can cause enteritis in pigs. A Danish study showed a significantly higher probability of detecting PCV2 without concurrent *L. intracellularis* infection, indicating that one of these pathogens has an impact on the dynamics of the other. Therefore, a delayed co-infection model was set up, initially aiming at investigating the interaction between PCV2 and *L. intracellularis* in pigs challenged with PCV2 and 2 weeks later with *L. intracellularis*. But due to PCV2 contamination of the *L. intracellularis* inoculum the aim was revisited to describing the infection dynamics and pathogenesis of pigs infected with PCV2 followed by delayed simultaneous exposure to PCV2 and *L. intracellularis*. Twenty-four high-health piglets were divided into three groups of eight pigs (A, B, C) and inoculated at experimental day (EXD) 0 with mock (groups A and B) or PCV2 (group C), and at EXD 14 with mock (group A) or *L. intracellularis*/PCV2 (groups B and C). The pigs underwent daily clinical examination, and were necropsied at EXD 51–52. Furthermore, histology, immunohistochemistry, serology and PCR for PCV2 and *L. intracellularis*, and measurement of C-reactive protein were carried out.

**Results:**

Group A remained negative for PCV2 and *L. intracellularis*. Following inoculation with *L. intracellularis*/PCV2, no significant differences were observed between group B and C, however pigs already infected with PCV2 (group C) showed milder clinical signs and exhibited milder intestinal lesions, less shedding of *L. intracellularis* and developed higher *L. intracellularis* antibody titers than the pigs in group B that only received the combined infection. Though the differences between group B and C were non-significant, all results pointed in the same direction, indicating that the pigs in group B were more affected by the *L. intracellularis* infection compared to the pigs in group C.

**Conclusions:**

Previous exposure to PCV2 had limited impact on the subsequent exposure to a combined *L. intracellularis*/PCV2 inoculation. However, there was a tendency that the infection dynamics of PCV2 and development of antibodies to PCV2 and *L. intracellularis* were altered in pigs previously exposed to PCV2. These differences should be confirmed in further experimental trials.

## Introduction

Porcine circovirus type 2 (PCV2) and *Lawsonia intracellularis* infections are both associated with unthriftiness, retarded growth, wasting, increased mortality and diarrhea in weaned and growing pigs (1–3). Both infections are ubiquitous in the pig population and cause substantial welfare problems and economic losses. PCV2 is a circular single-stranded DNA virus, which is recognized as the primary causative agent of postweaning multisystemic wasting syndrome (PMWS) and porcine circovirus-associated diseases (PCVAD) (4). Several disease complexes are classified as PCVADs, including PCV2-associated enteritis (1, 4, 5). *L. intracellularis* is an obligate intracellular Gram-negative bacterium which induces proliferative enteropathy mainly in the aboral small intestine. Proliferative enteropathy is characterized by thickening of the intestinal wall and mucosa, due to adenomatous proliferation of immature crypt epithelial cells and villous atrophy (2, 6, 7).

The exact role of PCV2 in the induction of enteritis is not clear, but studies have shown that PCV2 can induce granulomatous enteritis and lymphoid depletion and occasionally necrosis of the Peyer's patches and colon-associated lymphoid tissue (3, 8, 9). Thus, PCV2 infection may lead to a modulation of the local immune response, either paving the way for other enteric pathogens or even provide some kind of protection against subsequent infections. Indeed, PCV2 is known to interact with other pathogens and the immune system, and it is widely accepted that immunomodulation (e.g., concurrent infections, administration of immunomodulators) facilitate clinical manifestation of PCV2 infections (4, 10–12).

Intriguingly, a Danish field study on PCV2 and *L. intracellularis* induced enteritis, showed a significant higher probability of detecting PCV2 in pigs without a concurrent *L. intracellularis* infection – indicating a reciprocal relationship between the two pathogens (3). An experimental study with concomitant inoculation of PCV2 and *L. intracellularis* confirmed that enteritis can be induced by PCV2 alone, but showed neither differences in infection dynamics nor pathology in the co-infected group compared to the single inoculated groups (13).

Initially, the aim of the study was to investigate the interaction between PCV2 and *L. intracellularis* by setting up an experimental delayed co-infection model in naïve pigs where pigs were challenged initially with PCV2 and 2 weeks later with *L. intracellularis*. When the samples from the experiment were tested it was found that the *L. intracellularis* inoculum (enteric scrapings from naturally *L. intracellularis* infected pigs) was contaminated with PCV2 despite being immunohistochemically negative for PCV2. To avoid unnecessary waste of experimental animals we have decided to report the findings of the study by adapting the hypotheses and aims. PCV2 is present in the nursery section of many sow herds resulting in infection of pigs shortly after weaning, with development of PCV2 viremia at the age of 6–10 weeks (14, 15). In the Danish system, most pigs are transferred to specialized production herds at 30 kg body weight where they are mingled with pigs from other sources. Many of these production herds are positive for PCV2 and *L. intracellularis*, resulting in simultaneous re-exposure of the pigs to PCV2 and primary infection with *L. intracellularis*. It is tempting to hypothesize that early exposure to PCV2 could either attenuate or aggravate the severity of subsequent *L. intracellularis* infection. Thus, the revisited aim of the present study was to describe the infection dynamics and pathogenesis of pigs experimentally infected with PCV2 followed by simultaneous exposure to PCV2 and *L. intracellularis* 2 weeks later.

## Materials and methods

### Animals and experimental design

#### Animals

Twenty-four weaned, Landrace-Yorkshire piglets from three litters, from a small, non-vaccinated, high health herd (16), were allocated into three groups (A, B, C) of eight pigs each (Table 1). At experimental day 0, the piglets were 36 (litter I), 49 (litter II) and 53 (litter III) days old. To avoid any influence of age and/or body weight (i.e., adverse disease development) the piglets from litter I were allocated to the negative control group (group A). The average body weights at experimental day (EXD) −5 were 13.0 kg ± 4 g (group A), 19.2 kg ± 4 g (group B) and 19.4 kg ± 5 g (group C). Test of serum and feces samples from EXD 0 confirmed that the pigs were free from PCV2 and *L. intracellularis*. In addition, serological samples from EXD 0 and EXD 49 from all pigs were examined by routine diagnostic methods at the National Veterinary Institute and all samples tested negative for antibodies against porcine respiratory and reproduction syndrome virus (Type 1 and 2), influenza A (subtypes H1N1pdm09, avH1N1, avH1N2 and H3N2), *Actinobacillus pleuropneumonaie* (types 2 and 6), *Mycoplasma hyopneumoniae* and *Salmonella* sp.

**Table 1:** Experimental design of the delayed co-infection model.

**Group**	**Litter^a^**	**EXD^b^**	**0**	**14**	**50–51**
A	I (*n =* 6) II (*n =* 1) III (*n =* 1)		Mock^c^	Mock	End of experiment
B	II (*n =* 4) III (*n =* 4)		Mock	$L.in/PCV2^{c}$	
C	II (*n =* 4) III (*n =* 4)		PCV2^c^	L. in / PCV2	

^a^The groups originated from three litters of six (I), nine (II) and nine (III) piglets.

^b^Experimental day.

^C^The pigs were inoculated with porcine circovirus type 2 (PCV2), Lawsonia intracellularis and low amounts of PCV2 (L. in/PCV2) and/or Mock.

#### Experimental design

The three groups of pigs were housed in separate sections of the isolation facilities at the National Veterinary Institute, Lindholm. The pigs were allowed to acclimatize for 5 days before inoculation.

At EXD 0, the pigs in group C were inoculated with 10 ml of 1 x10^4.9^ TCID_50_ PCV2/ml; 6 ml of the inoculum was applied orally and 4 ml were given intranasally. In addition, the pigs in groups A and B were mock inoculated with a sterile PK15 cell culture in Eagle's medium by identical routes and volumes.

At EXD 14, the pigs in groups B and C were inoculated orally with approximately 8 x10^9^ *L. intracellularis* in 2 ml sucrose–potassium–glutamate buffer (SPG, pH 7.0) containing 2.4 x10^7^ PCV2 copies/ml as retrospectively determined by real-time qPCR. The pigs from group A were mock inoculated with sterile SPG buffer by identical route and volume. Oral inoculations were delivered by slowly dripping the inoculum (PCV2, *L. intracellularis*, mock) into the buccal cavity using a needle-free syringe.

The animal experiment was approved by the Danish Animal Experiments Inspectorate under the Ministry of Food, Agriculture and Fisheries. The animal care and used protocol adhered to the Danish law on animal experiments, which adheres to the EU directive on the protection of animals used for scientific purposes.

#### Inocula

The PCV2 inoculum was a 10th passage prepared from a cell culture isolate originating from a Danish PCV-2b field isolate from 2002 (16). The virus titer was 1 x10^4.9^ TCID_50_/ml Eagles medium. The inoculum contained 5.5 x 10^13^ PCV2 copies determined by real-time qPCR (17).

The *L. intracellularis* inoculum was produced according to the method described by Boesen et al. (18) with slight modifications from small intestinal sections from two naturally infected slaughter pigs with proliferative enteropathy. The intestines were culture negative for pathogenic *E. coli* and *Salmonella*, and immunohistochemically negative for PCV2 (see method below). On the day of inoculation, the homogenate was thawed at 4°C and further diluted in SPG. Examinations of ten-fold dilutions of the homogenate by indirect immunofluorescence (19) revealed a titer of the inoculum of 8.0 x10^9^ *L. intracellularis*. The inoculum was retrospectively tested for presence of PCV2 by real-time qPCR, revealing 2.4 x 10^7^ PCV2 copies/ml, i.e., 10^6^ PCV2 copies less than in the cell-grown primary PCV2 inoculum. These findings were consistent with the lower sensitivity of PCV2 immunohistochemistry compared with qPCR (20). It was not possible to isolate the PCV2 from the second inoculum in cell culture, and therefore the infectious titer expressed as TCID50 could not be determined.

#### Sampling procedures

Fecal samples were collected from the individual pigs on EXDs 0, 3, 7, 10, 14, 17, 21, 24, 28, 35, 42, and 50 (pigs no. 1–12) or 51 (pigs no. 13–24), and stored at −20°C until examined by real-time qPCR for presence of *L. intracellularis* and PCV2.

Blood samples were collected in vacutainers (Terumo Europe, Leuven, Belgium) from the anterior vena cava from all pigs for PCR and serological examinations. Non-stabilized blood samples were collected at EXDs 0, 3, 7, 10, 14, 17, 21, 24, 28, 35, 42 and 49. The samples were kept at 4°C for approximately 24 h before separation of serum, which was stored at −40°C for subsequent examination for PCV2 DNA, PCV2- and *L. intracellularis* antibodies and analysis of C-reactive protein (CRP).

### Clinical examinations and pathology

#### Clinical examinations

Individual pigs were subjected to daily clinical examination for signs of PCV2 and *L. intracellularis* infections. In order to obtain semi-quantitative measures for comparison of clinical disease between the three groups, a clinical scoring system modified after Mittelholzer et al. (21) was used. Clinical parameters characteristic for PCV2 and *L. intracellularis* infections were evaluated and scored on a 0–3 point scale, where 0 was regarded as normal and 3 represented severe symptoms of PCV2 or *L. intracellularis* infections. The highest obtainable score was 18 per pig/day. The parameters evaluated for each pig were: liveliness, appetite, signs of dehydration, skin color, respiration, and consistency of feces. Feces was also evaluated for odor, color and presence of blood. E.g., scores of fecal consistency were as follows: normal, solid feces (0); soft feces (1); loose, creamy feces (2); and watery feces (3). A comprehensive description of the clinical parameters to be scored is available in Supplementary File 1. Rectal temperatures were recorded for 3–4 days following PCV2 and *L. intracellularis* inoculations and if the pigs had reduced appetite or diarrhea. Temperatures of or above 40°C were regarded as fever.

The pigs were weighed on EXDs −5, 23, 36, and 49 and the average daily weight gain of the three groups was calculated.

#### Pathology and histology

At the end of the experiment on EXDs 50 (*n* = 12) and 51 (*n* = 12), the pigs were euthanized. Necropsy was performed for characterization of gross lesions, and tissue samples were collected for histological- and IHC examinations. The collected tissues included ileum (mid segment), cecum (apex), colon (apex of spiral) and ileocoecal lymph nodes. Furthermore, samples of lung, liver, kidney, tonsil, thymus, spleen, and bronchial- and superficial inguinal lymph nodes were collected and examined for signs of PMWS according to Opriessnig et al. (22). The samples were fixed in 10 % neutral buffered formalin and prepared for histology by routine methods, embedded in paraffin wax and sectioned at 3–5 μm. The sections were mounted on conventional glass slides and stained with haematoxylin and eosin (HE) for histological evaluation, or mounted on SuperFrost^®^ Plus slides (Mensel-gläser, Braunschweig, Germany) for IHC.

The histopathological examination was performed blinded and systematically. The following structures were evaluated in the intestinal tissue sections: villi in ileum, intestinal crypts, goblet cells, lacteals, and the intestinal associated lymphoid tissue (Peyer's patches). The mucosa in ileum, cecum and colon, lamina propria and submucosa were evaluated in regard to cellular density and -composition. The thickness of the colonic mucosa was examined by the use of Zeiss AxioVision software, version 4.7.1. (Carl Zeiss, Oberkochen, Germany). This was done by measuring the length of the crypts at 10 different locations of a HE stained tissue section at x25 magnification on a Zeiss Image M1 microscope (Carl Zeiss) and a mean length was calculated. If possible, the measurements were carried out on every 5th crypt being visible from top to bottom.

#### Immunohistochemistry

All samples of ileum, cecum, colon and the ileocoecal lymph nodes were examined for the presence of PCV2 capsid antigen by IHC, using an in-house mAb (F217) as previously described (3) and the same tissues were examined for presence *L. intracellularis* antigen by a previously described IHC method, using an in-house mAb (Law1-Dk) (18). Positive reaction for both IHC methods were scored semi-quantitatively as negative (0), low (1), moderate (2) or massive (3) and positive cells were morphologically characterized. For each pig a total PCV2- and *L. intracellularis* score was estimated based on the IHC scores of ileum, Peyer's patches, cecum, colon and the ileocoecal lymph node.

### Real-time qPCRs

DNA was extracted from serum and feces with QIAsymphony Virus/Bacteria Mini Kit (QIAGEN, Copenhagen, Denmark) and the levels of PCV2 and *L. intracellularis* were subsequently quantified in feces as previously described (23) and expressed as copies/g feces. The level of PCV2 in serum was quantified against a plasmid standard curve (17) and expressed as copies/ml serum.

### Examination of immune responses

Serum samples from EXDs 0, 7, 14, 21, 28, 35, 42, and 49 were examined by ELISA for antibodies to PCV2 (24) and *L. intracellularis* (25) using previously described methods.

The serum concentration of CRP at EXDs 0, 3, 7, 10, 14, 21, 24, 28, and 35 was analyzed by an indirect ELISA (26). The detection limit of the test was 708 ng/ml. The area under the curve (AUC) in a plot of CRP serum concentration against EXD was calculated for each pig for EXD 0–14 and EXD 14–35, respectively, using CRP concentration = 0 as the baseline.

### Statistical analyses

Pathologic data, clinical score, average weight, average daily weight gain, mean thickness of the colonic mucosa and IHC scores were analyzed by Mann-Whitney or Kruskall Wallis test using GraphPad Prism version 5.02 (GraphPad Software, San Diego, CA).

The levels of PCV2 and *L. intracellularis* DNA, and log-transformed antibody titers against PCV2 and *L. intracellularis* were compared groupwise for each sampling point using two-way ANOVA with Bonferroni post test performed using GraphPad Prism (version 4.00).

Mean AUC values for CRP of each group (A, B, C) were compared by Student's *t*-test using GraphPad Prism (version 5.02).

The statistically significant level was set at *P* < 0.05.

## Results

### Clinical examination and pathology

#### Clinical examination

**Figure 1:**
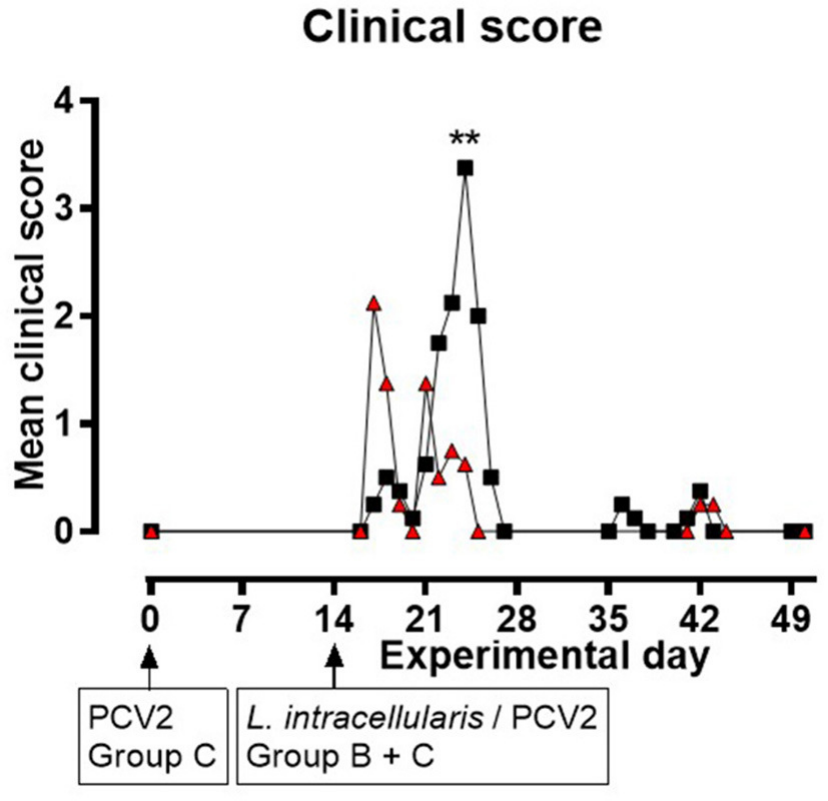
Mean clinical scores for groups B (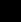) and C (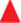) as a function of experimental day. None of the pigs in group A displayed clinical symptoms during the experiment (data not shown in this figure). Zero values are only presented at start and end of the study, and when next to a positive data point. Significant results (*p* < 0.01) are marked by **.

None of the pigs in group A displayed any clinical symptoms during the experiment. In groups B and C, changes in liveliness, appetite, and consistence, odor and color of feces were noted. Clinical signs related to intestinal disorders were observed from EXD 17 to 26 following inoculation with *L. intracellularis*/PCV2. All pigs in group B (8/8) and seven out of eight pigs in group C had reduced/soft fecal consistence for 1–5 days starting from EXD 17 to EXD 26. During EXD 22–25, 6/8 of the pigs in group B experienced non-profuse watery diarrhea lasting for 1 day (four pigs), 2 days (one pig) or 3 days (one pig), and they all had reduced appetite for 1–2 days, whereas in group C only one pig (1/8) had watery diarrhea (and normal appetite) on EXD 21. The daily clinical group score was increased among the pigs in group C during EXD 17–21 compared to the control pigs, whereas in group B the score peaked around EXD 22–25 (Figure 1). Generally, the score was higher in group B compared to group C, although the difference was only significant at EXD 25 (*P* < 0.01). There was a significant difference in cumulative clinical score for the period EXD 17–26 between the three groups (*P* = 0.0003), but the cumulative score between groups B and C was not significant (data not shown). Approaching the end of the study (EXD 36–43), one pig from group B and two pigs from group C were slightly depressed for 2–3 days. The numbers of pigs experiencing the various clinical signs during EXD 17–26 are listed in Supplementary File 1.

Only pigs in group B had rectal temperatures of or above 40°C. This was on individual days during EXD 15–18 (*n* = 1), EXD 21–22 (*n* = 2) and EXD 42 (*n* = 1), and one pig had increased temperature for two consecutive days (EXD 24–25).

The weights at EXD −5 were significantly different between the groups (*P* = 0.0050), due to the weights of group A being lower compared to group B (*P* = 0.0037) and C (*P* = 0.0028), whereas there was no difference between groups B and C. There were no difference in average daily weight gains between the groups (data not shown).

#### Gross pathology

The only lesion observed in the control pigs (group A) was a chronic infarct in the kidney of one pig. Among the remaining pigs, the main findings were related to the intestines and constituted of hyperemia of the gastric mucosa (group C: *n* = 2), hyperemia of one (group B: *n* = 4) or several (group C: *n* = 6) intestinal segments, thickening of the intestinal wall of jejunum and ileum (group B: *n* = 1), serosal edema of ileum and/or colon (group B: *n* = 1; group C: *n* = 1), liquid feces in colon (group B: *n* = 2) and increased volumes of clear abdominal fluid (group B: *n* = 2; group C: *n* = 4). The colon associated lymphoid tissue (group B: *n* = 2; group C: *n* = 3), ileocoecal lymph nodes (group B: *n* = 7; group C: *n* = 6) and mesenterial lymph nodes (group B: *n* = 2; group C: *n* = 3) were enlarged and/or edematous. Other findings were minor lung processes (group B: *n* = 2; group C: *n* = 1), mild interstitial lung edema (group C: *n* = 2), focally increased lobulation of the liver (group C: *n* = 2), enlarged superficial inguinal- (group B: *n* = 2) or bronchial (group C: *n* = 1) lymph nodes, and increased volumes of pericardial fluid (group C: *n* = 3). There were no obvious differences between group B and C in regard to frequency and severity of the lesions.

#### Histopathology

In the intestines and ileocoecal lymph nodes the main finding was infiltration of inflammatory cells. Only mild lesions were seen in a few pigs from group A. There were no obvious differences between group B and C in regard to frequency and severity of the lesions, except for the infiltration of neutrophils in the intestines and ileocoecal lymph nodes, which occurred more frequently among the pigs from group B, however the difference was not significant. The histological findings are summarized in Supplementary File 2.

The mean thickness of the colonic mucosa in group A was 264.5 μm ± 36.4, which was significantly thinner (*P* < 0.001) compared to the pigs in groups B (470.6 μm ± 75.9) and C (423.3 μm ± 65.5). There was no significant difference in the thickness of the mucosa between groups B and C.

There were no histological indications of proliferative enteropathy or PMWS in any of the pigs.

#### Immunohistochemistry

All control pigs (group A) were negative for PCV2 and *L. intracellularis* antigen by IHC. In groups B and C, PCV2 antigen was detected in macrophages in low to massive amounts in the examined tissues (Table 2). The pigs in group B obtained significantly higher total scores on PCV2 IHC compared to group C (*P* = 0.0014). In both groups, PCV2 was mainly detected in the lymphoid organs, i.e., the ileocecal lymph nodes and the Peyer's patches, however, several pigs in group B also had PCV2 positive macrophages in ileum, cecum and colon.

**Table 2:** Detection of porcine circovirus type 2 (PCV2) and *Lawsonia intracellularis* antigen by immunohistochemistry (IHC).

		**PCV2 IHC**						***L. intracellularis*** **IHC**			
**Group**	**Pig ID**	**Total score**	**Ileum^a^**	**PP^a^**	**cecum**	**colon**	**ln ileoc^b^**	**Total score**	**ileum**	**colon**	**ln ileoc**
A	1–8	0	0	0	0	0	0	0	0	0	0
B	9	1	0	0	0	0	1	0	0	0	0
	10	3	0	3	2	2	3	1	1	0	0
	11	2	0	2	0	1	1	1	1	0	0
	12	3	2	3	1	2	3	1	1	0	1
	13	2	0	1	0	0	2	1	1	0	0
	14	2	1	2	1	0	2	2	2	1	1
	15	2	1	2	0	0	2	1	1	0	0
	16	2	1	2	0	1	2	2	2	0	1
C	17	0	0	0	0	0	0	0	0	0	0
	18	1	0	1	0	0	0	2	2	0	1
	19	1	0	0	0	0	0	0	0	0	0
	20	1	0	0	0	0	1	1	1	0	0
	21	1	0	0	0	0	1	1	1	0	0
	22	1	0	1	0	0	1	1	1	0	1
	23	1	0	0	0	0	1	2	2	0	1
	24	1	0	0	0	1	1	1	1	0	0

Reaction for both IHC methods was scored as no staining (0), focal (1), moderate (2) or massive (3). There was no positive *L. intracellularis* IHC reaction in cecum or Peyer's patches.

^a^Ileum is divided into lymphatic tissue, i.e., Peyer's patches (“PP”) and “ileum” (ileum without PP) in regard to PCV2 IHC evaluation.

^b^Ileocecal lymph node.

*L. intracellularis* antigen was detected in low or moderate amounts in macrophages mainly in ileum and the ileocoecal lymph nodes in pigs from group B and C, however one pig (group B) also had low amounts of *L. intracellularis* in colon (Table 2). There was no significant difference between scores of group B and C with regard to *L. intracellularis* IHC. No PCV2 or *L. intracellularis* antigen was detected in enterocytes.

### Real-time PCR on feces and serum

**Figure 2:**
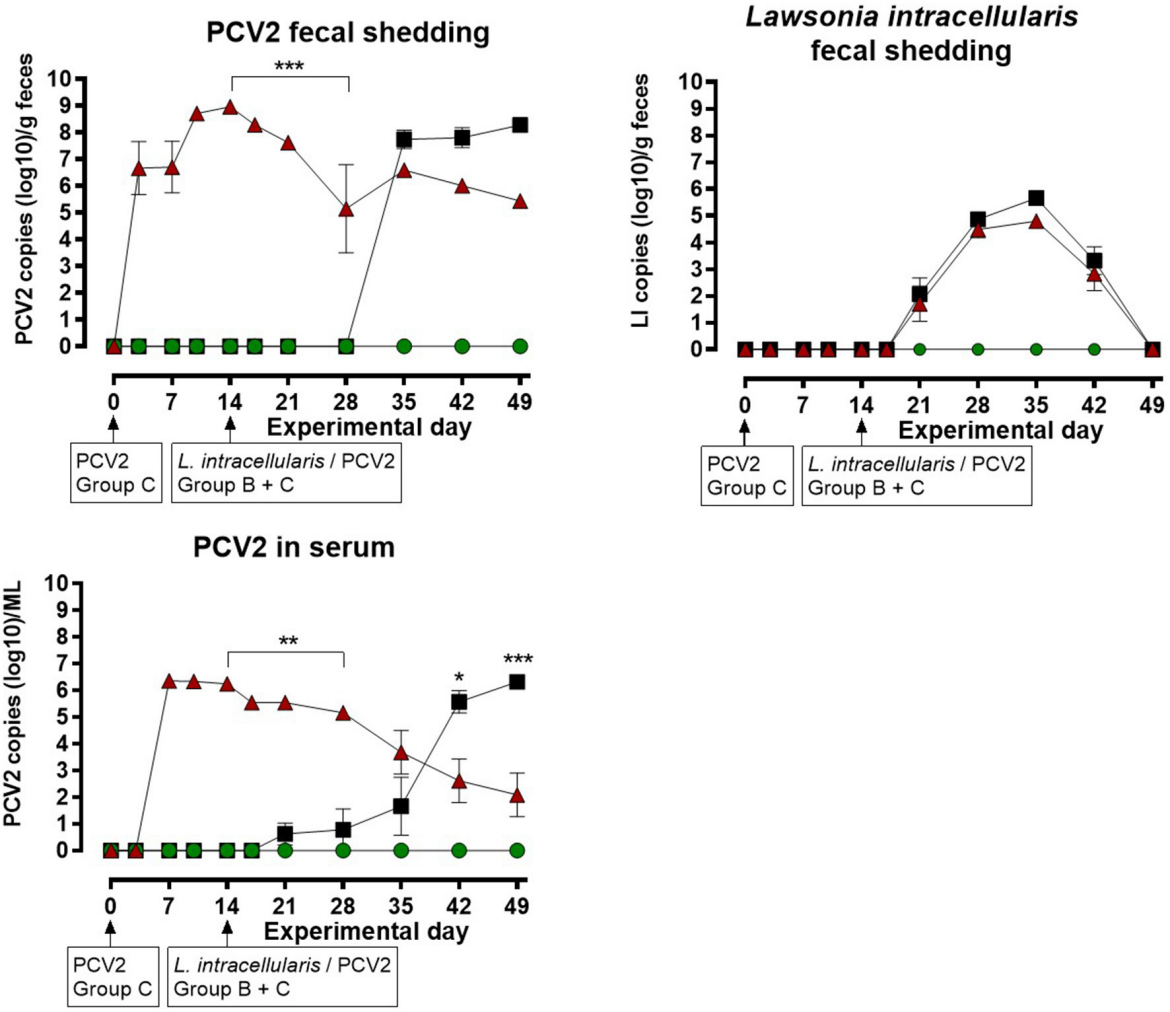
Porcine circovirus type 2 (PCV2) copies in feces and serum, and number of *Lawsonia intracellularis* in feces by quantitative PCR for the three groups: A (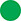), B (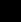), C (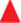) as a function of experimental day. Each dot represents mean ± SEM of eight pigs. Significant results (*p* < 0.05), (*p* < 0.01) and (*p* < 0.001) are marked by *, ** and ***, respectively.

The results of the quantitative PCV2 real-time PCR test on feces and serum are shown in Figure 2 for each of the three groups. The mock infected animals in group A were negative at all sampling points. Group C, infected with PCV2 on EXD 0, was positive for PCV2 in feces starting at EXD 3 in 7/8 pigs; reaching a maximum of 10^8^ copies/g feces at EXD 14, and thereafter a decline was seen until EXD 49. No increase in shedding of PCV2 was seen following the second exposure of these pigs at EXD 14. In group B, which was mock inoculated at EXD 0 and *L. intracellularis*/PCV2 inoculated at EXD 14, 8/8 pigs became PCV2 positive in feces 14 days post inoculation (EXD 35), reaching a maximum of 10^7.5^ copies/g feces at the last sampling day (EXD 49). The shedding of PCV2 in group C pigs was significantly higher than in group B between EXD 14–28 (*P* < 0.001), however, after EXD 35 there was no significant difference between groups B and C.

In group C, 8/8 pigs were positive for PCV2 in serum at EXD 7, where the maximum viral load of 10^6.5^ /ml was recorded, followed by a decline until the last sampling day (EXD 49). No increase in PCV2 load in serum was seen following the second PCV2 exposure at EXD 14. In group B, 2/8 pigs were weakly positive for PCV2 in serum at EXD 21 (7 days post inoculation), followed by at stepwise increase to maximum levels of 10^6^ /ml serum at EXD 49, where 8/8 pigs were positive. The serum level of PCV2 in group C pigs was significantly higher compared to group B from EXD 14–28, whereas the level of PCV2 was highest in group B pigs at EXD 42 (*P* < 0.05) and EXD 49 (*P* < 0.001).

The results of the *L. intracellularis* quantitative PCR test on feces are shown in Figure 2. The animals in group A were negative at all sampling points. *L. intracellularis* was detected in group B and C at EXD 21, reaching a maximum of 10^5^-10^6^ copies/g feces at EXD 35 and returning to below the detection limit at the termination of the study. The excretion of *L. intracellularis* among the pigs in group C was approximately one log lower at EXD 35 and 42 compared to group B, however, there were no significant differences in the level of *L. intracellularis* shedding between pigs from group B and group C at any sampling points.

### Examination of immune response

#### Antibodies to PCV2 and *L. intracellularis*

**Figure 3:**
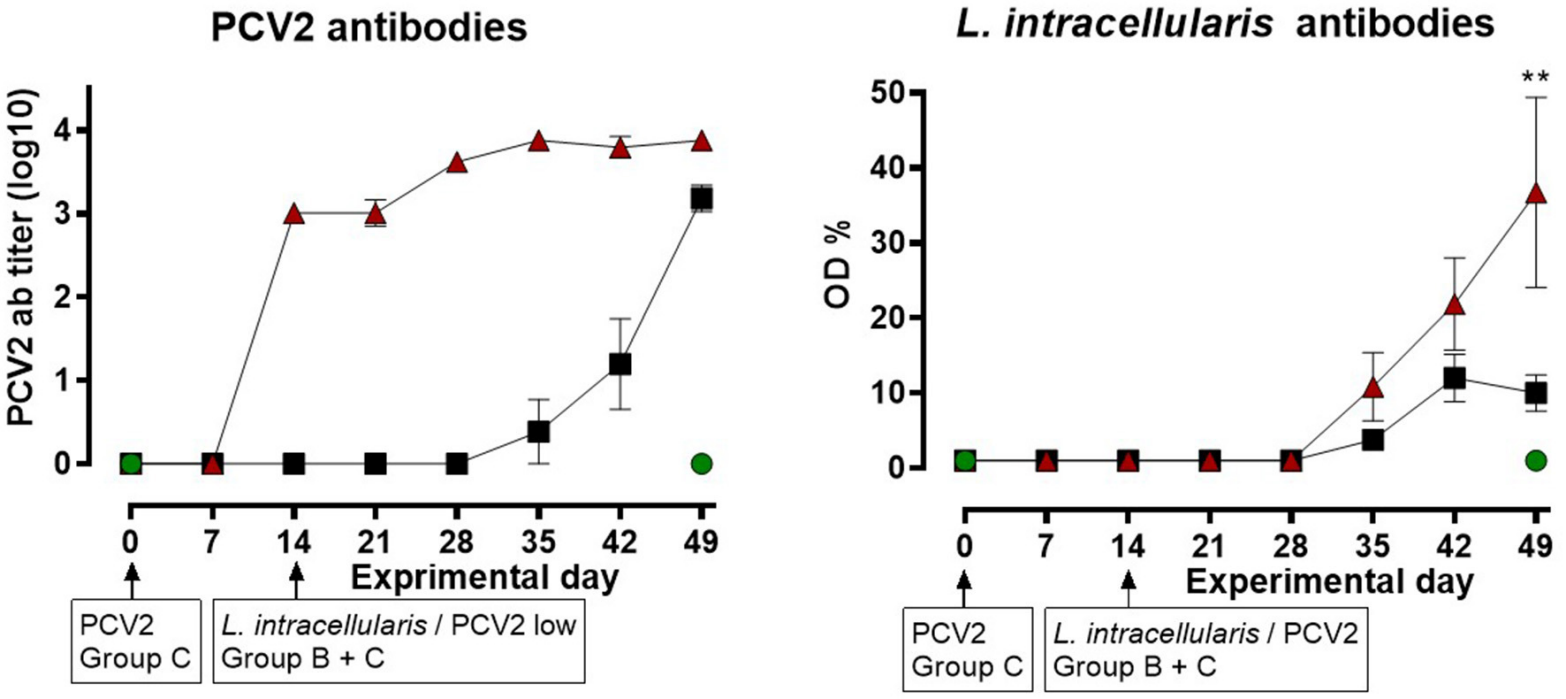
Serum levels of specific antibodies against Porcine circovirus type 2 (PCV2) (titer) and *Lawsonia intracellularis* (OD%), respectively by ELISA for the three groups: A (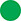), B (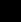), C (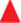) as a function of experimental day. Each dot represents mean ± SEM of eight pigs. Significant results (*p* < 0.01) are marked by **.

The pigs in group C, infected with PCV2 on EXD 0, developed high antibody titers against PCV2 at EXD 14 and the titers remained high throughout the study period – thus the pigs in group C had strong systemic humoral immune response to PCV2 when challenged with the *L. intracellularis*/PCV2 inoculum at PID 14 (Figure 3). Low levels of antibodies against PCV2 were also seen in group B at EXD 35, 21 days after exposure to PCV2, slowly rising to levels comparable to group C at EXD 49.

The pigs in groups B and C developed antibodies against *L. intracellularis* starting at EXD 35 (three weeks after *L. intracellularis* challenge). In group B, the titers remained low until termination at EXD 49, although a slight increase was observed between EXD 35 and EXD 42. Interestingly, the titers of *L. intracellularis* antibodies in group C were higher compared to group B, although this difference was only significant at EXD 49 (*P* < 0.01).

#### C-reactive protein

**Figure 4:**
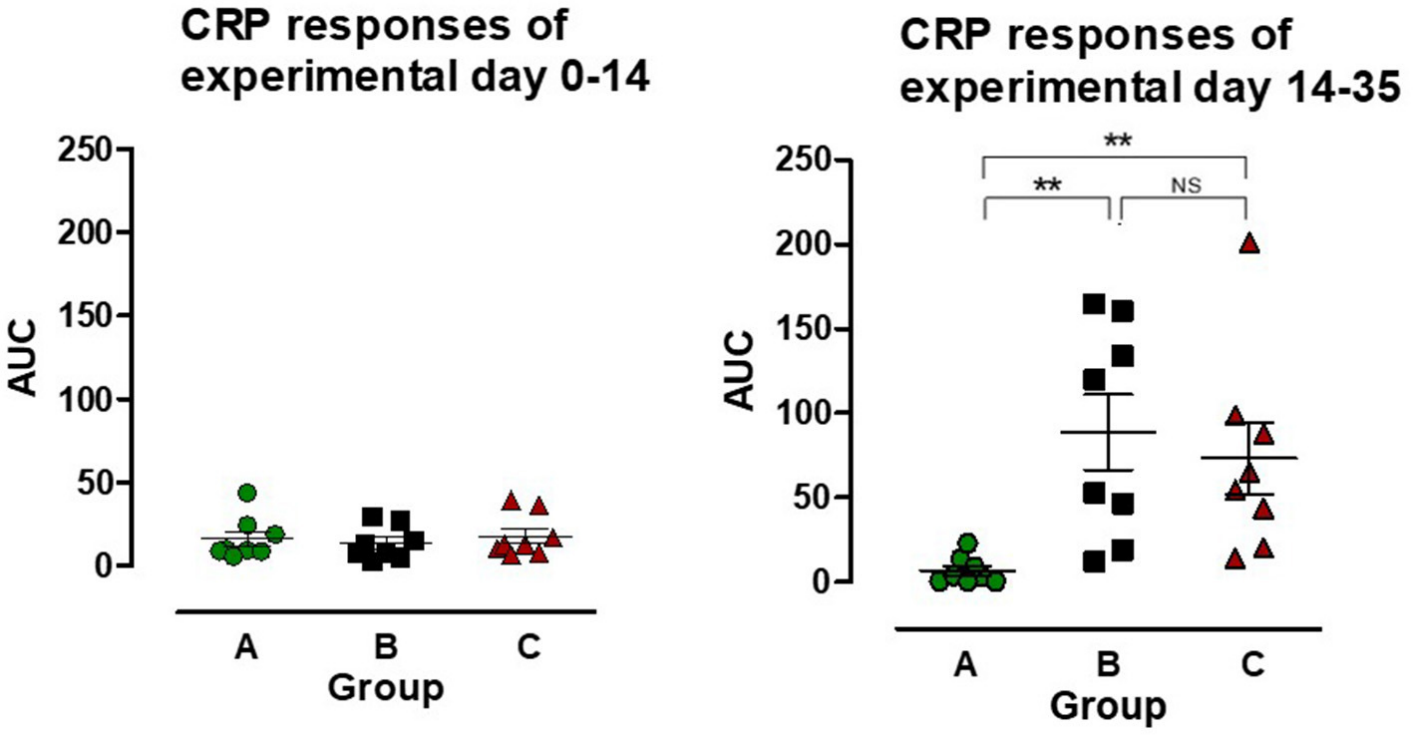
The area under the curve (AUC) for C-reactive protein (CRP) was calculated for individual pigs in the three groups: A (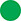), B (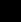), C (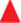) for the period after PCV2 inoculation (experimental day 0–14) and after *Lawsonia intracellularis*/PCV2 challenge (experimental day 14–35), respectively. Individual values as well as mean ± SEM for the group are shown. NS = non-significant. Significant results (*p* < 0.01) are marked by **.

PCV2 inoculation at EXD 0 did not lead to any change in CRP concentration, however after *L. intracellularis* infection there was a CRP response in both group B and C, resulting in a significant increase in AUC compared to the uninoculated control group (group A) (*P* < 0.01) (Figure 4). The CRP AUC values in group B at experimental day 14–35 were divided into two sub-groups of pigs having a low CRP response (*n* = 4) and a high CRP response (*n* = 4), but the responses were not significantly different between the two *L. intracellularis* inoculated groups.

## Discussion

Due to the unfortunate PCV2 contamination of the *L. intracellularis* inoculum, the study lacked an important control group of pigs solely inoculated with *L. intracellularis* and the results should therefore be interpreted having this limitation in mind. Nevertheless, the present experimental design reflect a situation that are common under typical pig production systems where pigs are often exposed to PCV2 in the nursery section at 6–10 weeks of age and then again to both PCV2 and *L. intracellularis* when they are moved to the finisher site at the age of 11–13 weeks, resulting in simultaneous re-exposure of the pigs to PCV2 and primary infection with *L. intracellularis*.

Several studies have examined the outcome of concurrent infections with PCV2 and other pathogens, focusing on the induction of PCVAD, the immunosuppressive role of PCV2 and/or factors that facilitate PCV2 replication (10, 11, 13, 16, 27, 28). In the present study, pigs of high sanitary status were initially exposed to PCV2 and subsequently to a combined *L. intracellularis*/PCV2 inoculum mirroring the situation often seen under field conditions (29–31). Similar to a previous study on concurrent inoculation of PCV2 and *L. intracellularis* (13), our study showed that prior exposure to PCV2 had no significant impact on the pathological responses to a subsequent combined *L. intracellularis*/PCV2 inoculation two weeks later. The PCV2 inoculation at EXD 0 did not induce clinical disease, which is in line with previous studies in high-health pigs (12, 32). The clinical responses to the combined *L. intracellularis*/PCV2 exposure at EXD 14 had a biphasic progress in group B and C pigs, and peaked on days 17–18 in group C and on days 22–25 in group B, with the pigs in the latter group displaying a higher maximum score. These differences were not related to differences in shedding of neither *L. intracellularis* nor PCV2 and may be co-incidental. The clinical signs were related to short episodes of watery diarrhea, which has been reported previously after *L. intracellularis* infection though uncomplicated, pastry diarrhea is more common (33, 34). The pigs had slightly reduced appetite, but were otherwise unaffected by the diarrhea and therefore no additional diagnostic stool examinations were carried out. Diarrhea has been demonstrated to appear approximately 9 days after infection with *L. intracellularis* (33). Therefore, the debut of diarrhea was expected to be around EXD 24 in our study, which correlated with the clinical findings in group B. Thus, the clinical scores together with the histopathology findings of increased neutrophilic intestinal infiltration (35) is likely to represent a typical response to a primary *L. intracellularis* infection of the pigs in group B. In a previous study with *L. intracellularis* mono-exposure, only few signs of diarrhea were encountered (18), whereas another study with combined *L. intracellularis*/PCV2 exposure in 1 month old pigs revealed development of bloody diarrhea at a later time point (21–28 days) after exposure (13). The explanation for the differences between the clinical responses of the two groups remains speculative. However, the reported immune modulating effect of PCV2 (12, 27, 36) may explain the early onset of diarrhea in group C caused by an increased intestinal susceptibility to the *L. intracellularis* infection due to the preceding PCV2 infection. However, the initial PCV2 infection did not induce an aggravated response to the subsequent *L. intracellularis* challenge, since the excretion of *L. intracellularis* and the acute protein responses were similar between the two infected groups. Pigs to PCV2 2 weeks prior to the combined *L. intracellularis*/PCV2 exposure, had a tendency to elicit a stronger antibody response to *L. intracellularis* compared to pigs only receiving a single exposure to PCV2, but the differences were only statistical different at EXD 49. Others (37, 38) detected development of antibodies already 2 weeks after inoculation with *L. intracellularis*, whereas Opriessnig et al. (13) and Cordes et al. (39) did not detect seroconversion until 3 weeks after exposure.

By immunohistochemistry, PCV2 antigen is mainly detected in macrophages, but can in some cases be detected in enterocytes (3), whereas *L. intracellularis* antigen is present in enterocytes up till 28 days post infection (33). The pigs were killed 36/37 days after exposure to the combined *L. intracellularis*/PCV2 inoculum correlating with detection of *L. intracellularis* antigen only in intestinal macrophages.

Following the initial PCV2 infection, the pigs in group C, developed viremia, started to excrete PCV2 in feces after 7 days, and seroconverted after 2–3 weeks, which are in accordance with previous findings (13, 16). In contrast, the pigs in group B did neither develop a marked viremia nor started to excrete virus until 2–3 weeks after infection and the seroconversion was delayed until 3–4 weeks after infection. These differences may be explained by the 6 log10 lower dose of PCV2 in the second inoculum, age differences between the pigs at the first and second exposures or may be due to interaction with the *L. intracellularis* in the second inoculum. Additional studies are needed to investigate this in further details.

C-reactive protein is a robust indicator of activation of the innate immune response in pigs by induction of high CRP serum concentrations following challenge with different pathogens (40–43). In our study, challenge with PCV2 at EXD 0 did not induce a CRP response corresponding with observations by Stevenson et al. (44), who demonstrated an increase in CRP levels among PMWS affected pigs, whereas no increase was seen in sub-clinically infected pigs. The challenge with *L. intracellularis* induced a low biphasic CRP response in both group B and C, i.e., a quick response on EXD 17, which lasted 3 days and a delayed response on EXD 35 (data not shown). A similar response to *L. intracellularis* infection has been reported previously (43). The CRP response of the pigs in group B tended to be higher and split into low- and high-responders, but the differences were non-significant and did not correlate with other parameters (clinical, pathological, antibody).

The results of the present study revealed that a previous exposure to high levels of PCV2 only had limited impact on the outcome of subsequent exposure to a combined *L. intracellularis*/PCV2 inoculation with most differences being non-significant. However, there was a tendency that the infection dynamics of PCV2 and the development of antibodies to PCV2 and *L. intracellularis* were altered in pigs previously exposed to PCV2. These differences may be accidental and should be confirmed in further experimental trials, preferably including a group of pigs inoculated with PCV2 only and groups infected with Lawsonia at specific, increasing time points after PCV2 inoculation in order to obtain a time line of the effect of PCV2 infection on the outcome of the Lawsonia infection.

## Data availability statement

The raw data supporting the conclusions of this article will be made available by the authors, without undue reservation.

## Ethics statement

The animal study was reviewed and approved by the Danish Animal Experiments Inspectorate under the Ministry of Food, Agriculture and Fisheries of Denmark.

## Author contributions

Experimental design was planned by LL, MH, JN, CH, and ØA. MH and JN carried out the animal experiment. Pathology (including histology and IHC) examinations were carried out by MH and TJ. The virology was prepared (inoculum), analyzed and interpreted by CH and LL. The bacteriology was prepared (inoculum), analyzed and interpreted by UR and ØA. PH analyzed and interpreted the CRP data. MH drafted the manuscript. All authors contributed to the article and approved the submitted version.

## Funding

The study was partly financed by the Boehringer Ingelheim European PCV2 award 2008.

## Conflict of interest

The authors declare that this study received funding from Boehringer Ingelheim (The European PCV2-award). The funder was not involved in the study design, collection, analysis, interpretation of data, the writing of this article, or the decision to submit it for publication.

## Publisher's note

All claims expressed in this article are solely those of the authors and do not necessarily represent those of their affiliated organizations, or those of the publisher, the editors and the reviewers. Any product that may be evaluated in this article, or claim that may be made by its manufacturer, is not guaranteed or endorsed by the publisher.
