# Supplementary material for: Experimental infection of high health pigs with porcine circovirus type 2 (PCV2) and Lawsonia intracellularis
Source: Front Vet Sci. 2022 Oct 6;9:994147. doi: 10.3389/fvets.2022.994147 (PMC9583870; doi:10.3389/fvets.2022.994147)
Supplement: Supplementary file 1 [file Table_1.docx]

**Supplementary file 1**

Total clinical group scores and number of pigs with clinical signs (above score 0) during experimental day (EXD) 17 to 26^1^.

|  | EXD | 17 |  | 18 |  | 19 |  | 20 |  | 21 |  | 22 |  | 23 |  | 24 |  | 25 |  | 26 |  |
| --- | --- | --- | --- | --- | --- | --- | --- | --- | --- | --- | --- | --- | --- | --- | --- | --- | --- | --- | --- | --- | --- |
|  | Group^2^ | B | C | B | C | B | C | B | C | B | C | B | C | B | C | B | C | B | C | B | C |
| Symptom^3^ | Total group  score | 2 | 17 | 4 | 11 | 3 | 2 | 1 | 0 | 5 | 11 | 14 | 4 | 17 | 4 | 27 | 5 | 16 | 0 | 4 | 0 |
| **Appetite** |  |  |  |  |  |  |  |  |  |  |  |  |  |  |  |  |  |  |  |  |  |
| Greedy, hungry | **0**^4^ |  |  |  |  |  |  |  |  |  |  |  |  |  |  |  |  |  |  |  |  |
| Eats slowly when fed | **1** |  | 2^5^ |  |  |  |  |  |  |  |  | 1 |  | 1 |  | 8 |  | 3 |  |  |  |
| Does not eat, shows no interest in food | **2** |  |  |  |  |  |  |  |  |  |  |  |  |  |  |  |  |  |  |  |  |
| **Fecal consistence** |  |  |  |  |  |  |  |  |  |  |  |  |  |  |  |  |  |  |  |  |  |
| Normal, solid | **0** |  |  |  |  |  |  |  |  |  |  |  |  |  |  |  |  |  |  |  |  |
| Soft | **1** | 1 | 5 | 4 | 4 | 3 |  | 1 |  | 3 | 5 | 4 | 4 | 1 | 1 |  | 2 | 5 |  | 4 |  |
| Loose, creamy | **2** |  |  |  | 1 |  |  |  |  | 1 | 1 |  |  |  |  | 1 | 1 | 1 |  |  |  |
| Watery | **3** |  |  |  |  |  |  |  |  |  | 1 | 2 |  | 3 |  | 3 |  | 1 |  |  |  |
| **Fecal odour** |  |  |  |  |  |  |  |  |  |  |  |  |  |  |  |  |  |  |  |  |  |
| Normal | **0** |  |  |  |  |  |  |  |  |  |  |  |  |  |  |  |  |  |  |  |  |
| Putrid | **1** | 1 | 5 |  |  |  |  |  |  |  |  | 2 |  | 3 |  | 4 | 1 | 2 |  |  |  |
| **Fecal colour** |  |  |  |  |  |  |  |  |  |  |  |  |  |  |  |  |  |  |  |  |  |
| Normal, brownish | **0** |  |  |  |  |  |  |  |  |  |  |  |  |  |  |  |  |  |  |  |  |
| Pale yellow, beige | **1** |  | 5 |  |  |  |  |  |  |  | 1 |  |  | 1 | 3 | 4 |  | 1 |  |  |  |
| Greyish | **1** |  |  |  | 5 |  | 2 |  |  |  |  | 1 |  | 2 |  |  |  |  |  |  |  |
| Dark, black | **2** |  |  |  |  |  |  |  |  |  |  |  |  |  |  |  |  |  |  |  |  |

^1^No symptoms occurred during EXD 0-16. The symptoms observed during EXD 27-51 were not related to the gastrointestinal tract and these are described in the text.

^2^None of the pigs in group A displayed any symptoms during the experiment, therefore only recordings from group B and C are included.

^3^Other symptoms looked for but not observed during EXD 17-26 were: **Liveliness** (Attentive, curious, awake, stands up immediately; Slightly depressed, stands up hesitantly without help; Depressed, gets up when forced, lies down again; Dormant, will not stand up), **Signs of dehydration** (No dehydration; Dehydrated, i.e. sunken eyes), **Skin colour** (Normal, light pink; Pale; Red-purple coloration), **Presence of blood in feces** (No visible blood; Small amounts of blood, i.e. a few bloody streaks; Moderate amounts of blood, i.e. distinct streaks/coagles; Much blood, feces is completely bloody) and **Breathing** (Normal steady breathing; Sneezes more than once; Coughs more than once; Labored breathing, i.e. distinct chest movement). However, none of these symptoms were observed during EXD 17 to 27.

^4^Symptoms were scored on a 0-3 point scale, where 0 was regarded as normal and 3 reflected a severe expression of that symptom.

^5^Number of pigs displaying the symptom.
