# Supplementary material for: Experimental infection of high health pigs with porcine circovirus type 2 (PCV2) and Lawsonia intracellularis
Source: Front Vet Sci. 2022 Oct 6;9:994147. doi: 10.3389/fvets.2022.994147 (PMC9583870; doi:10.3389/fvets.2022.994147)
Supplement: Supplementary file 2 [file Table_2.docx]

**Supplementary file 2**

Histological findings.

|  | | |  | Group | | |
| --- | --- | --- | --- | --- | --- | --- |
| Intestinal histopathology | | | Score | A | B | C |
| Diffuse or focal infiltration of mononuclear cells in lamina propria^1^ | | |  | **4** | **6** | **8** |
|  | | Ileum | + | *1* | *1* | *3* |
|  | |  | ++ |  | *3* | *1* |
|  | |  | +++ |  | *1* |  |
|  | | Cecum | + |  | *6* | *6* |
|  | |  | ++ |  |  | *1* |
|  | | Colon | + | *2* | *5* | *5* |
|  | |  | ++ | *1* | *2* | *2* |
|  | |  |  |  |  |  |
| Multifocal infiltration of neutrophils in lamina propria and/or intestinal crypts | | |  |  | **5** | **1** |
|  | | Ileum |  |  | *5* |  |
|  | | Cecum |  |  | *2* |  |
|  | | Colon |  |  | *2* | *1* |
|  | |  |  |  |  |  |
| Reduced number of goblet cells | | |  | **1** | **6** | **7** |
|  | | Ileum |  | *1* | *5* | *3* |
|  | | Cecum |  |  | *4* | *5* |
|  | | Colon |  |  | *3* | *1* |
|  | |  |  |  |  |  |
| Slightly atrophic villi of ileum | | |  |  | **4** | **3** |
|  |  | |  |  |  |  |
| Neutrophils in ileocoecal lymph nodes^2^ | | |  |  | **4** |  |

Bold numbers indicate the total number of animals with a lesion. Numbers in italics indicates lesions separated in intestinal compartments, where one pig can be represented in several intestinal segments. No finding is presented as blank.

^1^ The infiltration of cells were scored as few (+), moderate (++) or massive (+++). In ileum the cells mainly consisted of: lymphocytes and macrophages; in cecum: lymphocytes, plasma cells and some macrophages; and in colon: lymphocytes, macrophages and some plasma cells.

^2^ Diffuse infiltration or micro-abscesses.
